# miR-378a-3p Participates in Metformin’s Mechanism of Action on C2C12 Cells under Hyperglycemia

**DOI:** 10.3390/ijms22020541

**Published:** 2021-01-07

**Authors:** Ivo F. Machado, João S. Teodoro, Ana C. Castela, Carlos M. Palmeira, Anabela P. Rolo

**Affiliations:** 1 Department of Life Sciences, Faculty of Sciences and Technology, University of Coimbra, 3000-456 Coimbra, Portugal; imachado@cnc.uc.pt (I.F.M.); jteodoro@ci.uc.pt (J.S.T.); catycastela@hotmail.com (A.C.C.); palmeira@ci.uc.pt (C.M.P.); 2 CNC—Center for Neuroscience and Cell Biology, University of Coimbra, 3004-504 Coimbra, Portugal

**Keywords:** miR-378a-3p, metformin, mitochondria, mitophagy, C2C12

## Abstract

Metformin is the most used biguanide drug for the treatment of type 2 diabetes mellitus. Despite being mostly known for its hepatic anti-gluconeogenic effect, it is also known to modulate microRNAs (miRNAs, miRs) associated with metabolic diseases. The latter mechanism could be relevant for better understanding metformin’s mechanisms underlying its biological effects. In the current work, we found that metformin increases miR-378a-3p expression (*p* < 0.002) in C2C12 myoblasts previously exposed to hyperglycemic conditions. While the inhibition of miR-378a-3p was shown to impair metformin’s effect in ATP production, PEPCK activity and the expression of *Tfam*. Finally, mitophagy, an autophagic process responsible for the selective degradation of mitochondria, was found to be induced by miR-378a-3p (*p* < 0.04). miR-378a-3p stimulated mitophagy through a process independent of sestrin-2 (SESN2), a stress-responsible protein that has been recently demonstrated to positively modulate mitophagy. Our findings provide novel insights into an alternative mechanism of action of metformin involving miR-378a-3, which can be used in the future for the development of improved therapeutic strategies against metabolic diseases.

## 1. Introduction

Mitochondria are mostly known for their role as the main energy producers of most cells. Despite their role in aerobic respiration, they are also crucial for other important cellular processes, including cell metabolism regulation, calcium signaling and apoptosis [1]. Mitochondrial dysfunction can have a profound effect in the organism leading to failure of energy production, the excessive production of reactive oxygen species (ROS), and the disruption of metabolic homeostasis. Naturally, altered mitochondrial functioning is often implicated in metabolic diseases, such as type 2 diabetes mellitus (T2DM) and obesity, in neurodegenerative diseases, such as Alzheimer’s and Parkinson’s diseases, or in cancer [2,3].

Metformin (Met) has been demonstrated to have many beneficial effects in many diseases [4], being the most common drug for the treatment of T2DM, and is best known to decrease hepatic glucose production and to improve insulin resistance in both liver and skeletal muscle [5,6]. Metformin’s beneficial effects are well documented; however, its underlying mechanisms are still generating controversy. On the one hand, metformin inhibits mitochondrial complex I leading to the increase of cellular AMP: ATP ratio. In response to this energetic imbalance, AMP-activated protein kinase (AMPK) is activated, and through AMPK, metformin has been shown to improve mitochondrial function and increase mitochondrial biogenesis [7,8,9]. On the other hand, it was reported that even when AMPK is absent, metformin also has a glucose lowering effect [10]. These mechanisms of action are likely to be dependent on the administered concentration of metformin [11,12]. Nevertheless, increasing studies are reporting alternative AMPK-independent mechanisms of metformin involving microRNAs (miRNAs, miRs) [13,14], but few have succeeded in the establishment of a direct association between miRNAs and metformin’s mechanism of action.

MicroRNAs are small molecules composed of about 22 nucleotides that have been recognized as important post-transcriptional gene regulators, that can either inhibit messenger RNA (mRNA) translation or degrade mRNAs [15]. These small nucleotide sequences were shown to be important regulators of metabolic pathways, and it was demonstrated that miRNAs are also involved in mitochondrial metabolism [16]. Recently, miR-378a-3p, which is associated with metabolic diseases, was found to be upregulated by metformin [17]. miR-378a possesses two mature functional strands: a guide strand (miR-378a-3p) and a passenger strand (miR-378a-5p) [18]. Both miR-378a-3p and miR-378-5p are embedded in the *Ppargc1b* gene [19], which encodes PGC-1β, and both were implicated in the regulation of glucose and mitochondrial metabolism [18]. In fact, miR-378a-3p is highly expressed in tissues enriched in mitochondria, such as skeletal muscle, liver, and brown adipose tissue (BAT) [20,21]. miR-378a-3p was found to be upregulated in mice fed with a high fat-diet (HFD), being shown to regulate hepatic mitochondrial and fatty acid metabolism through carnitine-O-acetyltransferase (CRAT) [21]. Additionally, mice overexpressing miR-378a-3p/-5p were found to improve obesity by inducing brown adipose tissue expansion [22], and the overexpression of miR-378a-3p resulted in the improvement of systemic energy homeostasis [23]. The latter study concluded that miR-378a-3p improved systemic energy homeostasis by inducing the pyruvate-phosphoenolpyruvate (PEP) futile cycle in skeletal muscle [23]. In fact, skeletal muscle has a pivotal role in the maintenance of glucose and energy homeostasis being involved in a balanced crosstalk established between liver and adipose tissues [24]. *Ppargc1b* is highly expressed in skeletal muscle, and in this tissue, PGC-1β was shown to have a crucial role improving oxidative phosphorylation and inducing mitochondrial biogenesis in C2C12 cells [25]. The absence of PGC-1β led to impaired mitochondrial function and decreased skeletal muscle oxidative capacity [26]. Since *Ppargc1b* is the host gene of miR-378a-3p, it is suggested that miR-378a-3p may be involved in the regulation of the crosstalk between skeletal muscle and other tissues, as well as in the regulation of energy metabolism. Additionally, miR-378a-3p was also shown to maintain cell death programs in mice’s skeletal muscle, such as apoptosis and autophagy [20], suggesting a potential role for miR-378a-3p in selective mitochondrial degradation through mitophagy, which may contribute to the maintenance of a healthy mitochondria pool.

In the current study, we demonstrate that miR-378a-3p levels are increased by metformin in C2C12 myoblasts. When miR-378a-3p is inhibited, metformin’s biological effects are not observed, which suggests that miR-378a-3p is involved in the mechanism of action of metformin. In addition, miR-378a-3p was found to stimulate mitophagy in C2C12 cells exposed to hyperglycemia independently of sestrin-2 (SESN2).

## 2. Results

### 2.1. miR-378a-3p is Downregulated in C2C12 Myoblasts Exposed to Hyperglycemia-Mimicking Conditions

**Figure 1:**
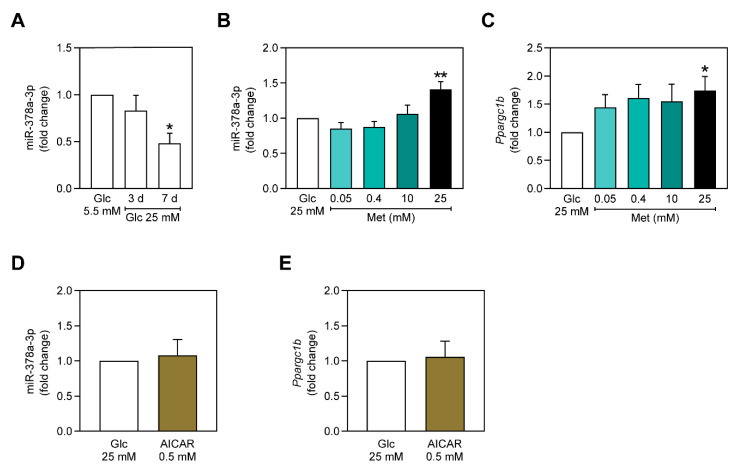
Higher concentrations of metformin upregulate miR-378a-3p and *Ppargc1b* in C2C12 myoblasts independently of AMPK. (**A**) miR-378a-3p expression in C2C12 cells after being exposed to 25 mM glucose for 3 and 7 days. (**B**) miR-378a-3p and (**C**) *Ppargc1b* expression in C2C12 myoblasts incubated with 0.05, 0.40, 10 and 25 mM of metformin. (**D**) miR-378a-3p expression and (**E**) *Ppargc1b* expression after incubation with 0.5 mM AICAR. All data is given as mean ± S.E.M. (*n* = 3). * *p* < 0.05, ** *p* = 0.0019 vs. Glc 25 mM.

In the past years, miR-378a-3p has emerged as a promising regulator of energy and mitochondrial metabolism, being shown to be involved in the regulation of metabolic disorders, such as T2DM and obesity [21,22,23]. Such disorders are often characterized by hyperglycemia-induced damage. Thus, to investigate the role of miR-378a-3p in metabolic unbalanced conditions, C2C12 cells were incubated with 25 mM of glucose for 3 and 7 days, after which miR-378a-3p expression was evaluated. As observed in Figure 1A, miR-378a-3p expression slightly decreases at 3 days of exposure to 25 mM of glucose and is significantly decreased after 7 days of hyperglycemia. Therefore, miR-378a-3p expression is only affected by long-term hyperglycemia exposure.

### 2.2. Metformin Upregulates miR-378a-3p and Ppargc1b Expression Independently of AMPK

Currently, metformin is the most used drug for the treatment of T2DM, being shown to improve hyperglycemic conditions. Nevertheless, it was also found to modulate many miRNAs that are associated with metabolic diseases [14]. For instance, miR-378a-3p was shown to be upregulated by metformin in HepG2 cells [17]. Thus, knowing that long-term hyperglycemia is associated with a decrease of miR-378a-3p expression (Figure 1A), we assessed if miR-378a-3p could be modulated by metformin before long-term hyperglycemia-associated metabolic stress arises. Therefore, we exposed C2C12 myoblasts to only 3 days of hyperglycemia and incubated them with increasing metformin concentrations for 24 h. Interestingly, while 0.05 mM, 0.40 mM and 10 mM of metformin did not affect miR-378a-3p expression, 25 mM of metformin upregulated miR-378a-3p (Figure 1B). These results suggest that miR-378a-3p expression is affected by metformin in a concentration dependent manner. Higher doses of metformin are required to induce miR-378a-3p transcription.

We next asked how metformin 25 mM could be inducing miR-378a-3p expression. It is known that miR-378a-3p is embedded in the first intron of *Ppargc1b*, which encodes the transcription factor PGC-1β, leading to the thought that miR-378a-3p is simultaneously transcribed with *Ppargc1b* [19,21]. Considering the previous studies, we hypothesized that metformin was inducing miR-378a-3p transcription through *Ppargc1b*. Indeed, only C2C12 cells treated with 25 mM of metformin were observed to have a significant increase in *Ppargc1b* (Figure 1C), thus showing that a higher concentration of metformin could be upregulating miR-378a-3p through *Ppargc1b*. Considering that metformin is able to activate AMPK and that this protein is tightly linked with PGC-1 [7,27], we evaluated whether AMPK could be responsible for the increase of miR-378a-3p expression. However, incubating C2C12 myoblasts, previously exposed to 3 days of hyperglycemia, with 5-aminoimidazole-4-carboxamide ribonucleotide (AICAR) 0.50 mM for 24 h had no effect on miR-378a-3p and *Ppargc1b* expressions (Figure 1D,E). This suggests that the upregulation of miR-378a-3p and *Ppargc1b* by metformin is independent of AMPK activation.

### 2.3. miR-378a-3p Is Required for Metformin-Associated Increase of Cellular ATP Content

**Figure 2:**
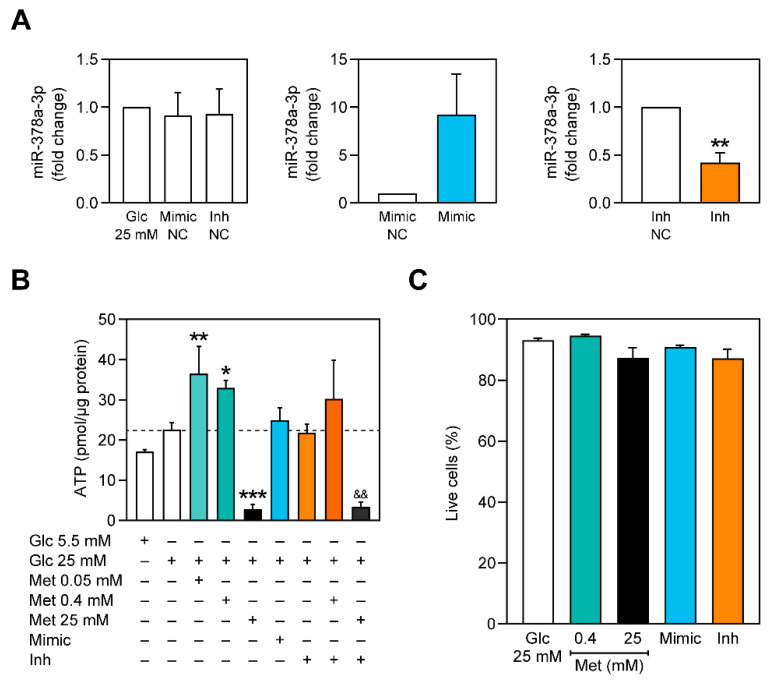
Lower concentrations of metformin increase ATP content in C2C12 myoblasts but this effect is impaired in cells with miR-378a-3p inhibited. (**A**) Expression levels of miR-378a-3p after transfection with mimic and inhibitor negative controls (Mimic NC and Inh NC, respectively), and with miR-378a-3p mimic and inhibitor in cells exposed to glucose 25 mM for 3 days. (**B**) ATP content in C2C12 myoblasts. (**C**) Cell viability assay using the Live/Dead viability/cytotoxicity assay kit. All data is given as mean ± S.E.M. (*n* = 3). * *p* < 0.05 versus Glc 25 mM, ** *p* < 0.01 versus Glc 25 mM, *** *p* = 0.0006 versus Glc 25 mM, && *p* = 0.0035 vs Inh.

A previous study showed that obese mice overexpressing miR-378a-3p had decreased ATP content in skeletal muscle due to the promotion of the pyruvate-PEP futile cycle [23]. Decreasing ATP in cells that have been exposed to hyperglycemic conditions could be beneficial due to the activation of energy-sensing proteins. When activated, such proteins act by placing in motion several mechanisms that stimulate mitochondrial biogenesis and mitophagy to counterbalance the energy deficiency, which can ultimately be beneficial for the cells. As such, we measured the content of ATP of C2C12 cells treated with metformin and transfected with miR-378a-3p mimics and inhibitors (Figure 2). As observed in Figure 2B, 0.05 mM and 0.40 mM of metformin significantly increased the ATP content of cells exposed to glucose 25 mM for 3 days, whereas a higher concentration of metformin decreased it. Neither miR-378a-3p overexpression nor miR-378a-3p inhibition affected ATP content of C2C12 myoblasts. However, the observed increase of ATP content by metformin 0.40 mM was abolished when miR-378a-3p was inhibited (Figure 2B), suggesting that miR-378a-3p is possibly involved in metformin’s mechanism.

Due to the verified depletion of ATP after treatment with 25 mM of metformin, a Live/Dead viability assay was performed to guarantee that treatments with metformin and transfections did not affect cell survival (Figure 2C).

### 2.4. PEPCK Activity Is Decreased with Increasing Concentrations of Metformin and miR-378a-3p Silencing

**Figure 3:**
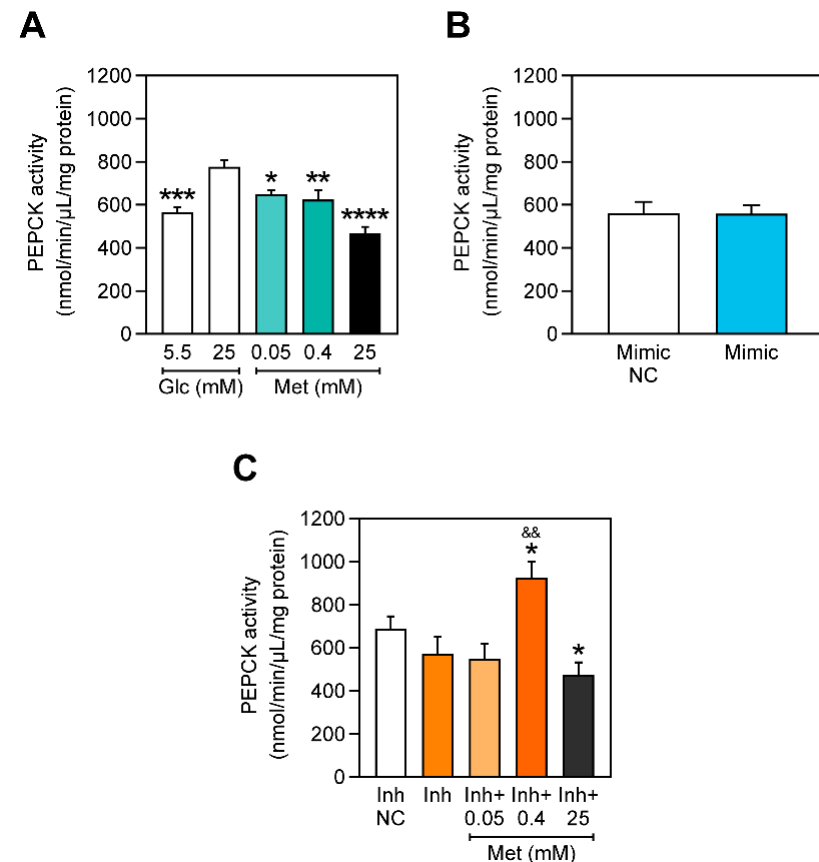
Metformin decreases PEPCK activity but not when miR-378a-3p is inhibited. PEPCK activity (**A**) in cells exposed to 0.05, 0.40 and 25 mM of metformin, (**B**) in cells overexpressing miR-378a-3p, and (**C**) in cells incubated with miR-378a-3p inhibitors and with 0.05, 0.40 and 25 mM of metformin. All data is given as mean ± S.E.M. (*n* = 3). * *p* = 0.0164 versus Glc 25 mM, ** *p* = 0.0052 versus Glc 25 mM, *** *p* = 0.0004 versus Glc 25 mM, **** *p* < 0.0001 versus Glc 25 mM, * *p* < 0.04 versus Inh NC, && *p* = 0.0038 versus Inh.

Metformin is extensively known for its hepatic anti-gluconeogenic effect [6,10,28]. PEPCK mediates the conversion of oxaloacetate into PEP and is one of the promoters of the energy-consuming gluconeogenic process. Hyperglycemia-mimicking conditions led to an increase of PEPCK activity, which, as expected, was reverted by metformin (Figure 3). miR-378a-3p was previously shown to ameliorate obesity in mice by stimulating the pyruvate-PEP futile cycle in skeletal muscle, which contributed to increased energy expenditure in this organ and consequently promoted inter-organ crosstalk with adipose tissue, improving the whole body homeostasis [29]. However, in our study, overexpressing miR-378a-3p in C2C12 myoblasts did not affect PEPCK activity, although inhibiting miR-378a-3p decreased PEPCK activity and impaired the PEPCK-lowering effect of metformin 0.40 mM.

### 2.5. Mitophagy Is Stimulated by miR-378a-3p

**Figure 4:**
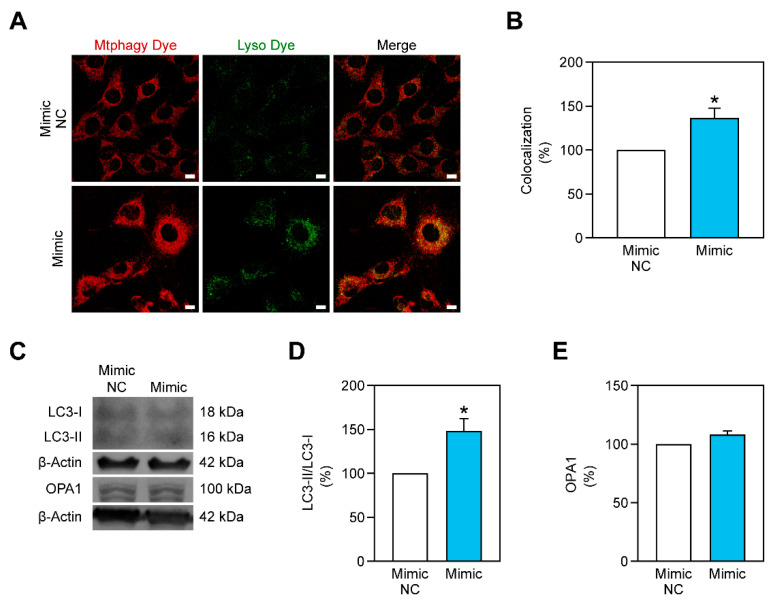
miR-378a-3p increases mitophagy in C2C12 myoblasts exposed to 3 days of glucose 25 mM. (**A**,**B**) Mtphagy dye (in red) and Lyso dye (in green) signals as well as the colocalization between them are increased when cells are transfected with miR-378a-3p mimic. Scale bar, 15 μm. (**C**) Representative images of Western Blot showing that the autophagy-related LC3-II/LC3-I ratio is increased for cells transfected with miR-378a-3p mimic, while mitochondrial fusion-related protein OPA1 was not altered. (**D**,**E**) Quantification of the LC3-II/LC3-I ratio and OPA1 protein content, respectively. All data is given as mean ± S.E.M. (*n* = 3). * *p* < 0.04 versus Mimic NC.

The maintenance of a healthy pool of mitochondria is guaranteed due to mechanisms of biogenesis and mitophagy. Mitochondrial function can be improved by stimulating such processes. During stress conditions, such as hyperglycemia, mitochondria are debilitated and the stimulation of mitochondria biogenesis or mitophagy could prove to be an efficient strategy to improve it. Recently, miR-378a-3p has arisen as a regulator of programmed cell death programs, such as autophagy and apoptosis, in skeletal muscle [20]. This study led us to hypothesize that miR-378a-3p can stimulate the selective degradation of damaged mitochondria through mitophagy and is capable of improving mitochondrial metabolism. To demonstrate this, we overexpressed miR-378a-3p in C2C12 cells and evaluated mitophagy using the Mtphagy Dye kit (Figure 4A). This method requires two different probes to successfully assess mitophagy: one targeting mitochondria (its fluorescence increases in an acidic environment) and another targeting lysosomes. The colocalization between the two probes allows the identification of mitochondria that are being degraded. Overexpressing miR-378a-3p in C2C12 myoblasts, previously exposed to 3 days of hyperglycemia, led to an increase of mitophagy (Figure 4A,B), and to an increase in the LC3-II/LC3-I protein ratio (Figure 4C,D). However, mitochondrial fusion-related protein OPA1 was not affected (Figure 4C,E).

**Figure 5:**
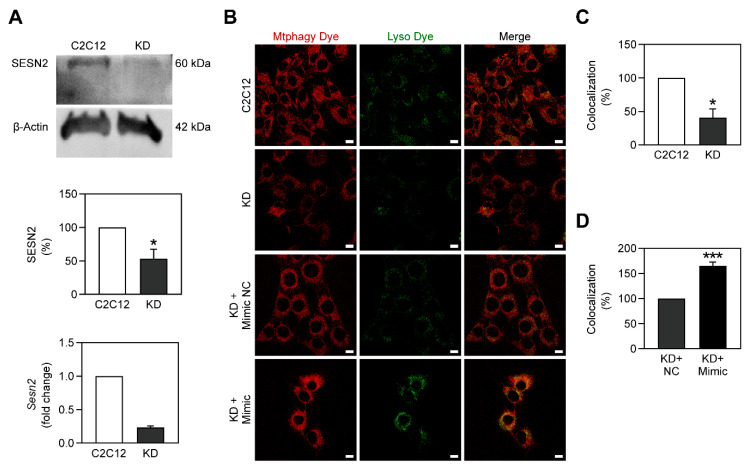
Mitophagy is diminished in *Sesn2* KD myoblasts and is rescued with the overexpression of miR-378a-3p. (**A**) Knockdown of *Sesn2* in C2C12 myoblasts was confirmed through Western Blot and RT-qPCR. (**B**–**D**) Mtphagy dye (in red) and Lyso dye (in green) signals as well as their colocalization are decreased when *Sesn2* is knocked down. Such parameters are increased when miR-378a-3p is overexpressed. Scale bar, 15 μm. All data are given as mean ± S.E.M. (*n* = 3). * *p* < 0.04 versus C2C12, *** *p* = 0.0009 versus KD + NC.

SESN2 is a protein that acts in response to metabolic stress by leading to the stimulation of several catabolic pathways [30]. More recently, SESN2 was identified as a crucial mediator of mitophagy, being involved in the PINK1-Parkin pathway [31]. To assess the relationship between miR-378a-3p and SESN2 in C2C12 cells, we knocked down the *Sesn2* gene, generating *Sesn2* KD C2C12 myoblasts (Figure 5A). As expected, the knockdown of *Sesn2* led to a decrease of mitophagy in C2C12 myoblasts (Figure 5B,C), reinforcing the role of SESN2 in mitophagy. Afterwards, *Sesn2* KD myoblasts were transfected with miR-378a-3p. The overexpression of this miRNA increased the fluorescence intensity of Mtphagy and Lyso dyes as well as their colocalization, suggesting that miR-378a-3p increased mitophagy in *Sesn2* KD cells (Figure 5B,D) and thus hinting that it improves mitophagy through a mechanism independent of SESN2.

### 2.6. Metformin Stimulates Tfam Expression only in the Presence of miR-378a-3p

**Figure 6:**
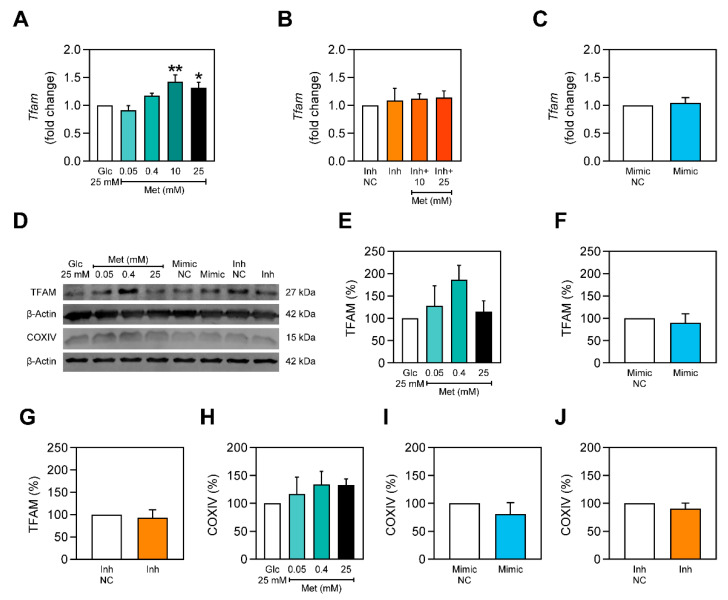
Metformin upregulates *Tfam* in C2C12 myoblasts but not when miR-378a-3p is inhibited. (**A**–**C**) Expression level of *Tfam* in C2C12 myoblasts. (**D**) Representative images of Western Blot showing TFAM and COXIV protein content. Mitochondria-related factors, such as (**E**–**G**) TFAM and (**H**–**J**) COXIV, were evaluated through Western Blot. All data is given as mean ± S.E.M. (*n* = 3). * *p* = 0.014 versus Glc 25 mM, ** *p* = 0.002 versus Glc 25 mM.

A healthy mitochondrial pool can only be maintained through a well-coordinated relation between mitochondrial biogenesis and mitophagy [32]. Metformin has been previously shown to stimulate mitochondrial biogenesis [7]. Since the effect of metformin was impaired by miR-378a-3p and miR-378a-3p was shown to improve mitophagy, we investigated whether it was capable of inducing mitochondrial biogenesis and whether it was also able to modulate the effect of metformin on mitochondrial biogenesis. This was verified through the evaluation of mitochondrial-biogenesis related factors such as TFAM and COXIV. C2C12 myoblasts exposed to 3 days of hyperglycemia were treated with metformin. qPCR revealed that *Tfam* was increased with metformin 10 mM and metformin 25 mM (Figure 6A), being impaired with the inhibition of miR-378a-3p (Figure 6B). Additionally, overexpression of miR-378a-3p did not increase *Tfam* (Figure 6C).

The protein content of TFAM was also assessed. TFAM tendentiously increases upon treatments with metformin but is not changed with either miR-378a-3p overexpression or silencing (Figure 6D–G). Additionally, COXIV content is not altered for either of the treatments (Figure 6H–J).

## 3. Discussion

MicroRNAs have not only been identified as relevant players in metabolic disorders but also as potential biomarkers for their detection [33,34,35]. For instance, miR-194, miR-148b, miR-223, miR-130a, miR-19a, and miR-1281 were found to be dysregulated in the plasma of patients with T2DM [33,36,37]. The non-invasive identification of microRNAs in circulation could prove to be crucial for the detection and assessment of T2DM. miR-378a has also been identified as a potential biomarker for metabolic disorders [35,38,39], and it arises as an important regulator of glucose and energy homeostasis [18,40]. In the current study, we evaluated the function of this specific miRNA in metabolic stress conditions using the immortalized mouse myoblast cell line C2C12. Skeletal muscle is known to actively participate in inter-organ crosstalk between adipose tissue and liver to maintain systemic energy homeostasis [24]. Therefore, studying novel regulators of skeletal muscle’s metabolism can prove to be effective for uncovering novel strategies to combat diseases resulting from metabolic and energetic dysregulation.

In the current study, we report that metabolic stress induced by exposure to high glucose media leads to a decrease of miR-378a-3p expression, but the period of exposure is crucial for this effect (Figure 1A). Different periods of exposure to hyperglycemia have been previously found to have different outcomes on cultured cells. HepG2 cells exposed for 7 days to hyperglycemia had their mitochondrial function impaired, whereas when exposed only to 3 days of hyperglycemia, no significant effect on mitochondrial function was observed [41]. Thus, we suggest that miR-378a-3p is sensitive to metabolic stress induced by prolonged hyperglycemia-mimicking conditions in C2C12 myoblasts. In other studies, miR-378a-3p expression is increased in response to starvation [20] and to fasting [42]. miR-378a-3p was also shown to be upregulated in the liver of high-fat diet (HFD)-fed mice while no alterations were observed in skeletal muscle [21]. Hence, this miRNA is likely to be modulated by diverse stress conditions, where its regulation is dependent on the particular tissue.

Hyperglycemia-related disorders are commonly found to be associated with metabolic dysregulation and mitochondrial dysfunctionality [43,44]. Dysfunctional mitochondria have defects in producing energy through the respiratory chain, which is associated with decreased oxygen consumption, higher ROS production and a decrease in ATP synthesis [45]. Metabolic disorders, such as T2DM, are commonly treated with metformin. Metformin’s mechanism of action remains controversial, although the existent evidence suggests that metformin’s concentration is crucial for its action. We observed that metformin inhibited ATP production at high concentrations, whereas at lower concentrations it stimulated ATP production (Figure 2B). Lower concentrations of metformin were also reported to increase ATP content, whereas higher concentrations were found to reduce it [8,46]. This is likely to be linked with the ability of metformin, at higher doses, to impair oxidative phosphorylation by inhibiting mitochondrial complex I [47]. Metformin is commonly known for its role in impairing gluconeogenesis [6]. PEPCK is one key enzyme that mediates gluconeogenesis and in agreement with previous studies, metformin decreased PEPCK activity (Figure 3A).

Lower doses of metformin did not have any effect on the expression of miR-378a-3p, but when a higher dose (25 mM) was administered, the expression of miR-378a-3p and *Ppargc1b* increased (Figure 1B,C). Similarly, higher doses of metformin (50 mM and 500 mM) were also reported to have a similar effect on the expression of miR-378a-3p in HepG2 [17]. The precise mechanism by which only a high dose of metformin upregulates miR-378a-3p is still to be fully understood. Even though we have not observed impaired cell viability for 25 mM of metformin and although metformin does not appear to induce apoptosis in C2C12 myoblasts [48], it was shown to impair cell proliferation [48]. Additionally, the time of exposure to metformin could also be a determinant factor, where a higher concentration of metformin could upregulate miR-378a-3p faster. Nevertheless, the mechanism of action of metformin seems to rely on the presence of this miRNA, as the effect of metformin 0.40 mM is impaired in cells that have miR-378a-3p inhibited. miR-378a-3p has been shown to be implicated in the regulation of glucose and energy metabolism and is described as an appealing target for the improvement of metabolic diseases [18,23,42]. Here, we demonstrate that in C2C12 myoblasts, neither ATP production nor PEPCK activity are affected by miR-378a-3p overexpression (Figure 2B and Figure 3). However, a previous study showed that miR-378a-3p overexpression increases energy expenditure in mice’s skeletal muscle through the activation of the Akt1/FOXO1/PEPCK axis, which resulted in the stimulation of the pyruvate-PEP futile cycle [23]. Surprisingly, the inhibition of miR-378a-3p impaired the effect of 0.40 mM of metformin in increasing ATP content (Figure 2B) and in decreasing PEPCK activity (Figure 3), indicating that miR-378a-3p is possibly implicated in the mechanism of action of metformin.

Mitochondrial function is assured due to processes of mitochondrial biogenesis and selective degradation of damaged mitochondria through mitophagy. Thus, a balance between those two processes is crucial to guarantee a healthy mitochondria pool in cells. miR-378a-3p has been reported to play a vital role in the maintenance of programmed cell death such as apoptosis [49,50] and autophagy [20]. Although, the role of miR-378a-3p in mitophagy was not verified. In the current work, we observed that miR-378a-3p overexpression stimulated mitophagy in C2C12 myoblasts exposed to hyperglycemic conditions (Figure 4). Mitophagy has been previously shown to be impaired during hyperglycemia [51], mitophagy-related proteins were reported to be decreased in T2DM human patients [52] and mitophagy-related genes were shown to be downregulated in the skeletal muscle of T2DM patients [53]. Hence, miR-378a-3p may have a crucial role promoting mitophagy in such conditions. However, this miRNA does not seem to promote mitochondrial biogenesis (Figure 6). Nevertheless, metformin was able to induce *Tfam* expression, despite only a non-significant increase in TFAM protein observed (Figure 6). Previous studies report that metformin promotes mitochondrial biogenesis through the activation of PGC-1α [7].

SESN2 is a protein that is sensitive to metabolic stress, being shown to be induced when cells are exposed to such stress [30]. This protein was reported to be a mediator of the PINK1-Parkin mitophagy pathway, being found to activate unc-51-like autophagy activating kinase (ULK1), and to consequently favor the translocation of Parkin into mitochondria [31]. miR-378a-3p is reported to promote autophagy through the activation of ULK1 [20]. Hence, to elucidate the mechanism by which miR-378a-3p activates mitophagy we used *Sesn2* KD myoblasts. These cells were found to have mitophagy impaired but miR-378a-3p was still capable of stimulating mitophagy in *Sesn2* KD cells (Figure 5). Considering the previous evidence, miR-378a-3p appears to induce mitophagy through a pathway involving ULK1 but independent of SESN2.

Here we advance a novel player in the mechanism of action of metformin in C2C12 myoblasts. Nevertheless, we acknowledge that the use of only one cell line is one limitation of our work. The use of another cell line would help to strengthen the discovered mechanism of action of metformin mediated by miR-378a-3p.

In conclusion, we show that miR-378a-3p is differentially expressed upon metabolic stress. Under such conditions, metformin’s beneficial effects are impaired by miR-378a-3p, suggesting that it is a player in metformin’s mechanism of action. Additionally, the overexpression of miR-378a-3p resulted in the increase of mitophagy, independently of the presence of SESN2, being likely to help maintain a healthy mitochondria pool in cells.

## 4. Materials and Methods

### 4.1. Cell Culture

C2C12 myoblasts, purchased from ATCC, were cultured in T75 flasks (Sarstedt, Nümbrecht, Germany) in 5.5 mM of glucose Dulbecco’s modified Eagle medium (DMEM; Sigma-Aldrich, St. Louis, MO, USA) supplemented with 10% fetal bovine serum (FBS; Invitrogen, Waltham, MA, USA) and 1% antibiotic–antimycotic (streptomycin/penicillin/amphotericin B; Gibco, Waltham, MA, USA), in a humidified 5% CO_2_ atmosphere at 37 °C. When cells reached 70–90% confluence, they were detached with TrypLE Express (Gibco, Waltham, MA, USA). Hyperglycemia conditions were mimicked by incubating cells with high glucose (25 mM) DMEM for 72 h. After 72 h of incubation, cells were seeded in multi-well plates and were treated with metformin (EMD Millipore, Burlington, MA, USA) for 24 h [54,55], or with AICAR 0.5 mM (Sigma-Aldrich, St. Louis, MO, USA).

### 4.2. Cell Transfection with miR-378a-3p Mimics and Inhibitors

C2C12 myoblasts were transfected with mirVana miRNA mimics and inhibitors (Invitrogen, Waltham, MA, USA) of mmu-miR-378a-3p (ACU GGA CUU GGA GUC AGA AGG), and mirVana miRNA Mimic Negative Control #1 (Invitrogen, Waltham, MA, USA) and mirVana miRNA Inhibitor Negative Control #1 (Invitrogen, Waltham, MA, USA) were used as the respective negative controls. Cells were transfected with the Neon Electroporation System (Invitrogen, Waltham, MA, USA) according to the manufacturer’s instructions. Briefly, after detachment with TrypLE Express, cells were washed with phosphate-buffered saline (PBS) 1X. The cells (1 × 10^5^) were resuspended in 10 μL of Resuspension Buffer R (Invitrogen, Waltham, MA, USA) and the miRNA sequence (25 μM) was added in order to reach a final concentration of 15 nM. A Neon Pipette (Invitrogen, Waltham, MA, USA) coupled to a 10 μL Neon Tip (Invitrogen, Waltham, MA, USA) was used to pipette the cell-miRNA mix that was submitted to 1 pulse of 1350 V with a width of 30 ms. After electroporation, cells were transferred to multi-well plates containing pre-warmed antibiotic-free media. After 24 h, the transfection efficiency was evaluated by RT-qPCR.

### 4.3. Generation of Sesn2 Knockdown Cells

C2C12 myoblasts were transduced using lentiviral particles containing pGIPZ plasmids (Dharmacon, Lafayette, CO, USA) with a sequence encoding a short hairpin RNA (shRNA) to target and silence *Sesn2*. All procedures were performed following the supplier’s instructions. The cells (1 × 10^5^) were seeded in multi-well plates and incubated overnight at 37 °C in a humidified 5% CO_2_ atmosphere. The next day, the medium was replaced by 1 mL of DMEM without FBS and without antibiotic–antimycotic but supplemented with viruses at a multiplicity of infection of 10. After 24 h, 1 mL of DMEM supplemented with 10% FBS and 1% antibiotic–antimycotic was added. The medium was replaced after 48 h with fresh medium containing puromycin 1 μg/mL so that cells that have efficiently incorporated the plasmid could be selected. The cells were cryopreserved until further use.

### 4.4. Cell Viability Assay

A Live/Dead Viability/Cytotoxicity kit (Invitrogen, Waltham, MA, USA) was used to evaluate cell viability. The supplier’s procedure was followed accordingly. Briefly, cells grown in multi-well plates were washed with PBS 1X, and 2 μM of calcein AM and 4 μM of EthD-1 were added. The cells were further incubated for 20 min at 37 °C. Then, they were visualized using a fluorescence microscope and images were taken. The number of live and dead cells was counted in Fiji [56,57].

### 4.5. RT-qPCR

Total RNA, including miRNAs, was extracted from C2C12 myoblasts using the miRNeasy Mini Kit (Qiagen, Hilden, Germany), according to the manufacturer’s protocol. The isolated RNA was quantified using a NanoDrop One (Thermo Scientific, Walhtam, MA, USA) instrument. The samples were stored at −80 °C until further use.

For quantification of miR-378a-3p, complimentary DNA (cDNA) was synthesized from 10 ng of total RNA using the TaqMan MicroRNA Reverse Transcription Kit (Applied Biosystems, Waltham, MA, USA) and according to manufacturer’s instructions. TaqMan Universal PCR Master Mix II (2X), no UNG (Applied Biosystems, Waltham, MA, USA) and TaqMan Small RNA Assays (Applied Biosystems, Waltham, MA, USA) were used for quantitative polymerase-chain reactions (qPCR), which were then read by the CFX96 Touch Real-Time PCR Detection System (Bio-Rad, Hercules, CA, USA).

For gene expression evaluation, cDNA was synthesized from 10 ng of total RNA and the iScript Advanced cDNA Synthesis Kit for RT-qPCR (Bio-Rad) was used according to the manufacturer’s instructions. SsoAdvanced Universal SYBR Green Supermix (Bio-Rad, Hercules, CA, USA) was used for qPCR reactions. The primers that were used are listed in Table 1. miRNA and gene expression were analyzed through the 2^−ΔΔCt^ comparison method, using 18S rRNA as housekeeping control.

### 4.6. ATP Content

ATP content was determined from C2C12 myoblasts grown in multi-well plates. In that sense, warmed PBS 1X was used to wash the cells that were then scraped in PBS 1X at 37 °C. Cells were centrifuged at 1000× *g* for 3 min and the pellets resuspended in 25 μL of KOH buffer (KOH 2.5 M, K_2_HPO_4_ 1.5 M) and 75 μL of H_2_O. Samples were sonicated and centrifuged at 18,000× *g* for 2 min at 4 °C. To the supernatants was added 100 μL of KH_2_PO_4_ 1 M and the pH was adjusted to 7. The samples were frozen in liquid nitrogen and stored at −80 °C until further use. The pellet was stored at −20 °C for protein quantification.

ATP Bioluminescent Assay Kit (Sigma-Aldrich, St. Louis, MO, USA) was used to quantify samples’ ATP, according to the supplier’s instructions. Bioluminescence was measured using a Victor^3^ plate reader (PerkinElmer, Waltham, MA, USA).

### 4.7. PEPCK Activity

PEPCK activity was assessed by using a PEPCK Activity Assay Kit (BioVision, Milpitas, CA, USA), according to the manufacturer’s instructions.

### 4.8. Western Blot

C2C12 myoblasts, grown in 60 mm petri dishes (Corning, Corning, NY, USA), were washed 4x with ice-cold PBS 1X, and were scraped in 1 mL of ice-cold PBS 1X. Cells were centrifuged for 3 min at 10,000× *g* at 4 °C, and the pellets were resuspended in ice-cold RIPA lysis buffer supplemented with protease inhibitors and Halt Phosphatase Inhibitor Cocktail (100X) (Thermo Scientific, Waltham, MA, USA). The samples were stored at −80 °C until further use.

Bicinchoninic acid protein assay was used to measure protein concentration. Protein was mixed with Laemmli buffer containing 8% β-mercaptoethanol and denatured at 80 °C for 5 min. Then, 50 μg of protein was loaded into an SDS-PAGE gel. Proteins were then transferred to a polyvinylidene difluoride (PVDF) membrane using a Trans-Blot Turbo Transfer System (Bio-Rad, Hercules, CA, USA). Membranes were blocked in 5% non-fat dry milk for 2 h and incubated with the primary antibodies at 4 °C, overnight. After washing membranes with TBS-T, they were incubated for 1 h with Biotin-XX goat anti-mouse or anti-rabbit from WesternDot 625 Western Blot Kits (Invitrogen, Waltham, MA, USA). Membranes were washed with TBS-T and were incubated with Qdot 625 streptavidin conjugate. Gel Doc EZ System (Bio-Rad, Hercules, CA, USA) was used to reveal the membranes. Images were analyzed using Image Lab 4.1 Software (Bio-Rad, Hercules, CA, USA). The antibodies that were used are listed in Table 2.

### 4.9. Mitophagy Detection

Mitophagy was evaluated in C2C12 myoblasts by the Mtphagy Detection Kit (Dojindo, Kumamoto, Japan), following the manufacturer’s instructions. Briefly, 2 × 10^4^ cells were seeded in μ-Slide 8 Well (ibidi, Gräfelfing, Germany) and treated as previously described. Following two washes with warm Hank’s Balanced Salt Solution (HBSS), they were incubated with 100 nM Mtphagy Dye. After 30 min of incubation, cells were again washed twice with warm HBSS and were further incubated with Lyso Dye for 30 min. Then, they were washed twice with warm HBSS and fluorescence was assessed using a Zeiss LSM 710 (Zeiss, Oberkochen, Germany) confocal point-scanning microscope. Images were analyzed in Fiji [56,57] and image colocalization was evaluated using a colocalization plugin algorithm (Pierre Bourdoncle, Institut Jacques Monod, Paris, France).

### 4.10. Statistical Analysis

Data are presented as mean ± S.E.M. Two-tailed student’s *t* test was performed to evaluate the statistical significance of two groups (*p* < 0.05) and one-way ANOVA was used for the evaluation of statistical significance of three or more groups (*p* < 0.05). Statistical analysis was performed using GraphPad Prism 8.0.2 (GraphPad Software, San Diego, CA, USA).

## Figures and Tables

**Table 1:** Nucleotide sequence of primers used in RT-qPCR.

Gene/miRNA	Primer Sequence (5′ to 3′)		NCBI Nucleotide’s Accession Number
mmu-miR-378a-3p	RT	TaqMan Assay 002243	-
	qPCR	TaqMan Assay 002243	
18S rRNA	Forward	GTA ACC CGT TGA ACC CCA TT	NR_046239.1
	Reverse	CCA TCC AAT CGG TAG TAG CG	
*Ppargc1b*	Forward	CTG GAA AGC CCC TGT GAG AG	NM_133249.3
	Reverse	CTG GAA AGC CCC TGT GAG AG	
*Sesn2*	Forward	TAG CCT GCA GCC TCA CCT AT	NM_144907.1
	Reverse	GAT TTT GAG GTT CCG TTC CA	
*Tfam*	Forward	CTT TGA GCC TTG ACA GAA G	NM_009360.4
	Reverse	ATA TGT AAC GGT CAT CAG TG	

**Table 2:** Primary and secondary antibodies used in Western Blot.

Antibody	MW (kDa)	Dilution	Supplier	Reference Number
*Primary antibodies*				
β-actin	42	1:2500	Sigma	A5441
COXIV	15	1:1000	Invitrogen	PA5-19471
LC3B	18, 16	1:1000	Sigma-Aldrich	L7543
OPA1	100	1:1000	BD Biosciences	612607
SESN2	60	1:100	Santa Cruz	sc-393195
TFAM	27	1:1000	Aviva Systems Biology	ARP36993
*Secondary antibodies*				
Biotin-XX goat anti-mouse IgG	-	1:2500	Invitrogen	W10132
Biotin-XX goat anti-rabbit IgG	-	1:2500	Invitrogen	W10142

## Data Availability

The data presented in this study are available on request from the corresponding author.

## Abbreviations

AICAR	5-aminoimidazole-4-carboxamide ribonucleotide
AMPK	AMP-activated protein kinase
BAT	Brown adipose tissue
CRAT	Carnitine-O-acetyltransferase
HFD	High-fat diet
Met	Metformin
miRNA	MicroRNA
PEP	Phosphoenolpyruvate
PEPCK	PEP carboxykinase
PGC-1	PPARγ coactivator-1
PPARγ	Peroxisome proliferator-activated receptor-γ
ROS	Reactive oxygen species
SESN2	Sestrin-2
shRNA	Short hairpin RNA
T2DM	Type 2 diabetes mellitus
TFAM	Mitochondrial transcription factor A
ULK1	Unc-51 like autophagy activating kinase
